# Automated detection of over- and under-dispersion in baseline tables in randomised controlled trials

**DOI:** 10.12688/f1000research.123002.1

**Published:** 2022-07-12

**Authors:** Adrian Barnett

**Affiliations:** 1 Australian Centre for Health Services Innovation & Centre for Healthcare Transformation, Queensland University of Technology, Kelvin Grove, Queensland, 4059, Australia

**Keywords:** automation, randomisation, trials, fraud, reporting errors, Bayesian analysis

## Abstract

**Background**: Papers describing the results of a randomised trial should include a baseline table that compares the characteristics of randomised groups. Researchers who fraudulently generate trials often unwittingly create baseline tables that are implausibly similar (under-dispersed) or have large differences between groups (over-dispersed). I aimed to create an automated algorithm to screen for under- and over-dispersion in the baseline tables of randomised trials.

**Methods**: Using a cross-sectional study I examined 2,245 randomised controlled trials published in health and medical journals on *PubMed Central*. I estimated the probability that a trial's baseline summary statistics were under- or over-dispersed using a Bayesian model that examined the distribution of t-statistics for the between-group differences, and compared this with an expected distribution without dispersion. I used a simulation study to test the ability of the model to find under- or over-dispersion and compared its performance with an existing test of dispersion based on a uniform test of p-values. My model combined categorical and continuous summary statistics, whereas the uniform uniform test used only continuous statistics.

**Results**: The algorithm had a relatively good accuracy for extracting the data from baseline tables, matching well on the size of the tables and sample size. Using t-statistics in the Bayesian model out-performed the uniform test of p-values, which had many false positives for skewed, categorical and rounded data that were not under- or over-dispersed. For trials published on *PubMed Central*, some tables appeared under- or over-dispersed because they had an atypical presentation or had reporting errors. Some trials flagged as under-dispersed had groups with strikingly similar summary statistics.

**Conclusions**: Automated screening for fraud of all submitted trials is challenging due to the widely varying presentation of baseline tables. The Bayesian model could be useful in targeted checks of suspected trials or authors.

## Introduction

Papers describing the results of a randomised trial often include a table that compares the randomised groups at baseline (hereafter called a “baseline table”). This baseline table presents summary statistics that describe the groups, such as average age and the percentage of males. The table’s purpose is to demonstrate that the randomisation produced similar groups, which strengthens the case that any differences between groups are due to the randomised treatment. ^1^ A baseline table is recommended by the CONSORT guidelines, which were designed to improve the reporting of randomised trials. ^2^

Researchers who fabricated randomised trials have been discovered because their baseline tables were not realistic. ^3^^–^^5^ When fabricating the baseline table they created highly comparable groups that would pass peer review. In trying to avoid raising alarms during peer review, they unwittingly raised an alarm at post peer review. Fraudulent researchers might also create baseline data with unusually large differences between groups, likely because they do not understand how to create realistic summary statistics when data are truly random. ^6^ Fraudulent researchers may not be uncovered by one baseline table alone, but an odd table might prompt a wider investigation. ^7^

Fraudulent researchers have so far been found in ad hoc ways, including concerns being raised by whistleblowers and researchers noticing strange patterns whilst reading papers in their field or conducting systematic reviews. ^8^^,^^9^ Other problems have been found by dedicated researchers trawling through papers. Manually extracting data from papers is time consuming and automatic data extraction would be a useful advance. ^10^ Automated detection algorithms would save time and increase scrutiny. ^11^^,^^12^

Previous statistical methods for finding problems in baseline tables have used the p-values from tests comparing groups at baseline, and then tested if the distribution of p-values is uniform. ^13^^,^^14^ However, it is possible to get a non-uniform distribution of p-values when the two groups were randomised, for example, for skewed data. ^15^ Another limitation with this approach is that it can only use summary statistics of the mean and standard deviation for continuous variables, so summary statistics using percentages are not included. ^16^^,^^17^ This is a large loss as percentages are commonly used in baseline tables. I aimed to create a method that could use summary statistics from both continuous and categorical variables.

The aim of this paper is not to provide undeniable evidence for fraud. Baseline tables that appear to have a problem could occur for a range of non-fraudulent reasons. These include planned or unplanned factors to do with randomisation, such as dynamic randomisation to create highly comparable groups, or subversion of the random allocation. ^18^ Problems can also be due to mislabelled summary statistics or reporting errors. ^11^^,^^14^ My aim was to create an automated algorithm that could be used to flag potential problems at the submission stage, which would help researchers improve their paper prior to publication. ^19^

## Methods

I report *PubMed Central* ID numbers to highlight examples without citing papers. The example baseline tables can easily be examined by interested readers using the *PubMed Central* site (see extended data).

There are two parts to the methods and results: 1) The new Bayesian test for under- or over-dispersion, 2) The automated extraction of baseline tables.

### Baseline tables

An example of a baseline table from a randomised trial is shown in Table 1. The table compares the continuous variable of age using the mean and standard deviation, and the categorical variable of gender using numbers and percents.

**Table 1.** Example of a baseline table comparing three randomised groups at baseline. This is illustrative data and not from a real trial.

	Treatment group		
	A ( *n* = 20)	B ( *n* = 21)	C ( *n* = 22)
Age, mean (SD)	17.1 (4.4)	18.2 (3.8)	16.5 (3.3)
Gender, Male, n (%)	10 (50)	10 (48)	10 (45)
Female, n (%)	10 (50)	11 (52)	12 (55)

The key idea of this work is to use the summary statistics to examine if there is under-dispersion (the statistics are too similar) or over-dispersion (the statistics are too different) given that the data are from a randomised trial. I combined continuous and categorical or binary summary statistics from the baseline table by summarising the difference between randomised groups using the independent samples t-statistic. It may be surprising to use the t-statistic to compare categorical data like gender, but the t-test is robust in situations where the chi-squared test would be a common choice, even for small sample sizes. ^20^

I excluded rows from the baseline table that were the inverse of the previous row, for example the percentage male followed by the percentage female. In this case the t-statistics for males and females would be perfectly negatively correlated and including these results twice would artificially increase the sample size. I excluded rows where the t-statistic was the inverse of the previous, but not where the t-statistic was zero. This approach only excludes rows that are a perfect inverse and would miss other grouped results, such as rows for three age groups. The effect of including these correlated table rows are examined in the simulation study.

I did not use summary statistics that were the median and quartiles or minimum to maximum, as I could not compare these statistics using the parametric t-test.

I created t-statistics for all pairs of comparisons. For example, for the three-group trial in Table 1 there would be three comparisons: A vs B, A vs C, B vs C.

### Bayesian model of observed differences

The observed differences ( *d*) in the summary statistics of randomised groups were modelled using a t-distribution.

$$d_{i,j}∼t\left(0,\sigma_{i,j}^{2},{\mathrm{df}}_{i}\right),\;i=1,\ldots ,T,j=1,\ldots ,n_{i},$$

where *i* is the trial index and *j* is the row in the table. The expected mean difference is zero, which should be the case for randomised groups.

The pooled inverse-variance is the precision and was modelled as

$$\sigma_{i,j}^{-2}=s_{i,j}^{-2}\times \gamma_{i},$$

where $s_{i,j}^{2}$ is the reported pooled variance. The trial-level random variable *γ _i_* was used to model a difference in the precision for trial *i* using a spike-and-slab approach ^21^:

$$\log\left(\gamma_{i}\right)=\left[\left(1-P_{i}\right)\times 0\right]+\left[P_{i}\times \epsilon_{i}\right],$$

$$P_{i}∼\text{Bernoulli}\left(0.5\right),$$

$$\epsilon_{i}∼N\left(0,10\right).$$

Each trial had a “switch” *P _i_* ∈ (0, 1), that determined whether it is part of the spike or slab. The spike at zero, with *P _i_* = 0, was for trials where the differences between randomised groups were as expected ( $\sigma_{i,j}^{-2}=s_{i,j}^{-2}$ ). The slab, with *P _i_* = 1, was for trials with under- or over-dispersion. This difference was modelled using a normal distribution where over-dispersed results have a negative *ϵ _i_* (and multiplier under 1, *γ _i_* < 1) and under-dispersed results have a positive *ϵ _i_* ( *γ _i_* > 1). The variance for this normal prior of 10 is small compared with typical vague priors in Bayesian models, but in preliminary modelling I found this covered the full range of possibilities, including where the summary statistics were identical between randomised groups, and variances larger than 10 caused convergence issues. The binary switch for each trial ( *P _i_*) was modelled using a Bernoulli distribution.

For continuous data I used the difference in group means and pooled variance as follows (dropping the *i* and *j* subscripts for simplicity):

$$d={\bar{x}}_{1}-{\bar{x}}_{2},$$

$$s^{2}=\frac{\left(1/n_{1}+1/n_{2}\right)\left[\left(n_{1}-1\right)s_{1}^{2}+\left(n_{2}-1\right)s_{2}^{2}\right]}{\left(n_{1}+n_{2}-2\right)},$$

where ${\bar{x}}_{g}$ is the mean, *s _g_* the standard deviation, and *n _g_* the sample size in group *g.* For categorical data I used:

$$p_{1}=r_{1}/n_{1};\;p_{2}=r_{2}/n_{2},$$

$$d=p_{1}-p_{2},$$

$$s^{2}=\frac{\left(n_{1}-1\right)p_{1}\left(1-p_{1}\right)+\left(n_{2}-1\right)p_{2}\left(1-p_{2}\right)}{\left(n_{1}+n_{2}-2\right)},$$

where *r _g_* is the numerator and *n _g_* is the denominator for group *g.*

The degrees of freedom (df) for trial *i* is the total sample size minus one ( *n*_1_ + *n*_2_ − 1), which allows for greater variance in differences for smaller trials.

Two statistics can be used to judge whether a baseline table has under- or over-dispersion:

• The estimated trial-specific probability of under- or over-dispersion ${\bar{P}}_{i}=\sum_{j=1}^{M}P_{i}^{\left(j\right)}/M$ , for which I examined a threshold of ${\bar{P}}_{i}>0.95$ to flag a potential problem by averaging over the *M* Markov chain Monte Carlo estimates.
• The estimated precision ${\bar{\epsilon }}_{i}$ which indicates larger under- or over-dispersion for values further from zero. This is also averaged over the Markov chain Monte Carlo estimates.

### Established problematic trials

To examine how the method performed for trials that are very likely fraudulent, I used the trials published by Yuhji Saitoh which were identified as problematic by Carlisle and colleagues. ^22^ I extracted the baseline tables for the first ten trials in date order and examined how the evidence of under- or over-dispersion accumulated over time. For comparison, I calculated the existing uniform test of p-values. ^13^ I calculated the statistics using continuous summary statistics only, and for combining continuous and categorical summary statistics.

### Simulation study

I used a simulation study to examine differences between the new Bayesian method using the t-distribution and the existing method using p-values and the uniform distribution. I simulated data using two scenarios described by Bland ^15^ with no concern about randomisation but where the p-value distribution would be non-uniform, meaning the uniform test could return a high percentage of false positives:

• Small trials with a sample size of 10 and summary statistics using binary data (e.g., percent of males)
• Large trials with a sample size of 1,000 and summary statistics using skewed continuous data (e.g., length of hospital stay)

As a comparison with the small binary scenario, I added a large binary scenario which would be expected to have more uniform p-values due to the larger sample size:

• Large trials with a sample size of 1,000 and summary statistics using binary data

To examine the power of my Bayesian method to detect problematic tables, I used three additional scenarios that used a 50:50 mix of binary and continuous summary statistics and where the underlying data were:

• Under-dispersed: randomised groups were too similar. Achieved by copying half of the means and percentages from one group to the other.
• Over-dispersed: randomised groups were too different. Achieved by adding a large number to the group means or percentages.
• As expected for a randomised trial.

To create realistic tables, simulation parameters were based on a large sample of baseline table data from my automated extraction applied to *PubMed Central* (see extended data). These parameters were: the ratio of continuous:binary summary statistics and the distributions of group sample sizes and table rows. The group sample sizes were randomly generated using an exponentiated gamma distribution with shape of 11.2 and rate of 3.0, which gives a median sample size of 37 and first to third quartile of 19 to 83. The number of rows per baseline table were randomly generated using a gamma distribution with shape 2.2 and rate 0.15, which gives a median number of rows of 12 and first to third quartile of 7 to 19.

Summary statistics in baseline tables are often rounded, and hence I rounded the mean to one decimal place and the standard deviation to 2 decimal places. To examine stronger rounding I rounded the mean to zero decimal places and a standard deviation to 1 decimal place. Rounded statistics could create under-dispersion by concealing the differences between groups.

To examine a scenario where the uniform test should perform well, I used a simulation with no dispersion, all continuous summary statistics, and means rounded to 3 decimal places.

To examine the effect of including multi-group categorical data (e.g., low, middle, high income), I simulated categorical data with three groups.

For all scenarios I created 500 simulated trials and each trial had two randomised groups with equal sample size. I compared the statistics graphically using distributions of the p-values and t-statistics. For the existing uniform test, I tested if the p-values for each study followed a uniform distribution using the Kolmogorov–Smirnov test, and counted the number of simulations where the null hypothesis was rejected using the 0.05 threshold. Using my Bayesian model, I examined the number of trials where the estimated probability of under- or over-dispersion ( $\bar{P}$ ) was higher than 0.95.

### Automated extraction of baseline tables

To create a large and generalisable sample of baseline tables, I extracted tables from the National Library of Medicine’s *PubMed Central* open access subset which has 3.7 million papers. The steps are outlined below and the complete code is available on *GitHub.* ^23^

I downloaded a list of published randomised trials from the *trialstreamer* web page ^24^ using the *PubMed Central* ID (PMCID). The *trialstreamer* data was downloaded on 9 August 2021 and had 57,109 trials with a PMCID. For logistical reasons I reduced the full list to a random sample of 10,000.

I next accessed the available papers from the open access subset using the PMCID. All available papers were download in XML format and read into R.

I excluded papers that were not randomised trials, including: i) trial protocols, and ii) papers that re-used trial data for other study designs (e.g., diagnostic accuracy). This exclusion was made based on the title and abstract, but some protocols were not identified in the title or abstract and hence were wrongly included.

The algorithm searched the full text for the baseline table using key words and phrases in all table captions. These words included “baseline”, “characteristic” and “demographic”. I also searched for words and phrases in the caption that ruled out baseline tables, such as “drug information” and “change from baseline”. The key words and phrases were found using trial and error.

If a baseline table was found, then I extracted: all the summary statistics, the type of summary statistics (e.g., median or percentage), and the group sample sizes. A challenging step was estimating what summary statistics were used in each row of the baseline table. This was estimated based on the text in the rows and columns (e.g., “Mean”, “%”, etc) and the variable label, as some variables such as age and BMI were often continuous, whereas other variables such as gender were categorical.

A key step was estimating the groups’ sample sizes. These were first estimated by searching the column and row headers for key indicators such as “N=”. If no samples sizes could be found, then they were estimated from all available percentages in the baseline table. These estimated sample sizes were only used if there was a strong agreement, defined as an inter-quartile range of less than 1. For example, estimated sample sizes from four percentages of 65, 65, 65 and 64, would be acceptable and would use the mode of 65. A paper was excluded if the sample sizes could not be extracted.

### Predictors of under- or over-dispersion

I applied my Bayesian model to all the baseline tables extracted from *PubMed Central* to give the probability of under- or over-dispersion for each trial. I then examined whether there were study design characteristics associated with the probability of under- or over-dispersion. I used a multiple linear regression model with a dependent variable of the study-level probability of a non-zero dispersion ( $0\leq {\bar{P}}_{i}\leq 1$ ), and independent variables that described the paper, study design and features of the table. I included independent variables of the journal and country of first author, using a combined “other” if a journal had fewer than 10 trials and a country fewer than 20 trials. For the study design, I included if the study was a pilot (based on the title), a cluster-randomised trial (based on the title and abstract), or used the standard error of the mean instead of the standard deviation (based on the baseline table). Features of the baseline table included as predictors were the number of rows, number of columns, sample size, largest difference in sample size between groups, proportion of continuous summary statistics and average number of decimal places for summary statistics. I selected a smaller subset of key predictors from the larger set using the elastic net as a variable selection tool. ^25^

### Estimation

All the *R* code to extract the tables and run the Bayesian model is openly available https://github.com/agbarnett/baseline_tables. ^23^ An interactive version of my Bayesian model is available via *shiny*: https://aushsi.shinyapps.io/baseline/. The Bayesian models were fitted using *WinBUGS* Version 1.4.3 ^26^ for the paper and *nimble* version 0.12.1 ^27^ for the shiny application. The Bayesian models used two chains with a burn-in of 2,000 followed by 2,000 samples thinned by 2. The data management and plotting were made using *R* version 4.1.1. ^28^

### Ethical considerations

I used publicly available data that were published to be read and scrutinised by researchers and hence ethical approval was not required.

## Results

### Established problematic trials

**Figure 1.**
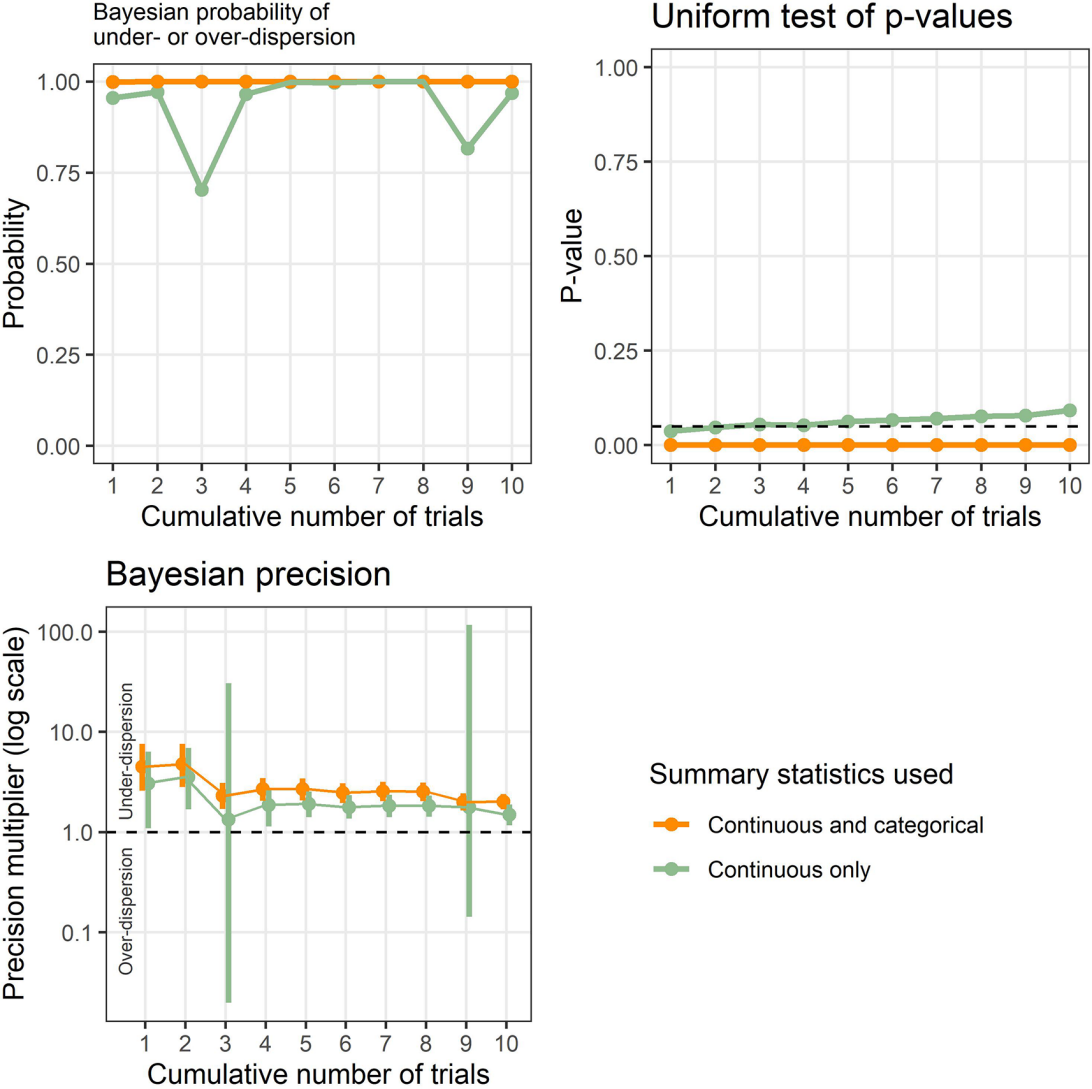
Results from the Bayesian model and uniform test of p-values applied to a set of known problematic trials. The results are cumulative to highlight the effect of accumulating evidence over time.

Example results applied to known problematic trials are shown in Figure 1, and show the new Bayesian method and existing test based on the uniform distribution. The results are cumulative to show the effect of accumulating evidence, which was 3 table rows in trial 1 up to 38 table rows for trials 1 to 10. Ten (26%) of these rows were categorical summary statistics.

The Bayesian probability of under- or over-dispersion ( ${\bar{P}}_{i}$ ) using continuous and categorical data is 1 from the first trial and remains at 1 for all ten trials, strongly signaling an issue with the tables. The Bayesian probability just using continuous data is relatively high, but dips when trials 3 and 9 are added. The p-value from the uniform test is zero across all ten trials, strongly signaling an issue with the tables. However, the p-value gradually increases with accumulating trials when using only continuous summary statistics, and was 0.09 when using all 10 trials.

The Bayesian precision shows that the summary statistics are under-dispersed and the 95% credible intervals narrow with accumulating evidence. However, when using only continuous summary statistics, the intervals widen greatly when including trials 3 and 9 and the intervals include potential over-dispersion.

### Simulation results

The results for the nine simulated scenarios are in Table 2. The uniform test performed well for the simulations that used continuous data with minimal rounding or large binary data, with rejection rates close to the expected 5%. However, it had high false positive percentages for the other five scenarios where there was no under- or over-dispersion, doing particularly badly for small binary data and skewed data. The uniform test did have good power to detect under- and over-dispersed data. Examples for single simulations are shown in the extended data and show the non-uniform distribution when the data are skewed, small binary or rounded.

**Table 2.** Results for the simulation study. Percentages of simulated trials that were not flagged or were flagged as under- or over-dispersed using a > 0.95 probability threshold, and a test of uniformity for the p-value distribution using a < 0.05 threshold.

Simulation type	Uniform test rejected (%)	As expected/Under-dispersed/Over-dispersed (%)
*Simulated data that is not under- or over-dispersed*		
Continuous, minimal rounding	6.0	100/0/0
Large binary	5.6	99.6/0.4/0
50:50 binary:continuous	18.6	99.8/0/0.2
Rounded	21.2	97.0/0/3.0
Correlated categorical	27.4	97.4/2.0/0.6
Small binary	31.6	98.4/1.6/0
Skewed	43.8	98.8/1.0/0.2
*Simulated data that is under- or over-dispersed*		
Over-dispersed	74.6	13.6/2.0/84.4
Under-dispersed	95.0	84.0/16.0/0

The Bayesian model rarely flagged trials where there was no under- or over-dispersion, hence there were few false positives. The Bayesian model was successful at detecting trials that were over-dispersed, with 84.4% of the simulations flagged at the 0.95 threshold. The model was less successful at detecting trials that were under-dispersed, with 16.0% of the simulations flagged as under-dispersed at the 0.95 threshold.

### Validation of automated table extraction

I validated my algorithm to extract baseline tables using manually-entered baseline data from randomised trials. To find eligible trials I searched *PubMed* for randomised trials between 2017 and 2020 that were available open access on *PubMed Central* and were not protocols, which gave 25,760 trials. A random selection of 200 trials was made, with the results compared between the algorithm and manually entered data. 118 papers were excluded by the algorithm, with the three most common reasons because the paper was not openly available ( *n* = 48), there was no baseline table ( *n* = 36), or there was no comparison between groups in the table ( *n* = 16). A further 9 papers could not be compared because the manually entered data were judged not to be randomised trials. This left 73 baseline tables to compare.

Detailed comparisons of the algorithm and manual results are in the extended data. In summary, my algorithm correctly determined the summary statistic 87% of the time (795 out of 909; 95% CI: 85% to 90%). The biggest differences were when the algorithm wrongly chose continuous for a median (2%) or could not chose any statistic when the row was a percentage (5%). The algorithm was able to extract the sample size, with a mean difference of 0 (5% to 95% percentile: 0 to 0). The algorithm accurately estimated the size of the baseline table, with a median difference of 0 (5% to 95% percentile: –1 to 4) for the number of rows, and 0 (5% to 95% percentile: 0 to 0) for the number of randomised groups.

The accuracy of the algorithm is reasonable given the large variety in the presentation of baseline tables, an issue that has been flagged by others. ^29^ Failures of the algorithm sometimes meant the table data were excluded as the algorithm could not extract the numbers, this means that I was not able to completely screen the literature. Failures when the wrong data were extracted, for example wrongly extracting a total column as a randomised group, sometimes led to the trial being flagged by my model and I examine this issue in the next section.

### Results for *PubMed Central* trials

The majority of the 10,000 potential trials were excluded ( Table 3). The three most common reasons were:

• there was no baseline table or one could not be detected by my algorithm,
• the XML file was not available despite being on the open access *PubMed Central* database,
• it was not a randomised trial.

**Table 3.** Number of excluded trials and reasons for *PubMed Central* trials.

Reason	n	Percent
No baseline table	2824	37.4%
Full text page not available	2127	28.2%
Not a randomised trial	1547	20.5%
No sample size	408	5.4%
Just one column in table	304	4.0%
Follow-up results in baseline table	162	2.1%
Just one sample size	72	1.0%
Pre-post comparison	43	0.6%
Single-arm study	43	0.6%
Atypical table layout	14	0.2%
Could not detect statistics	9	0.1%
Total	7553	100.0%

There were 2264 included trials with a total of 72,090 table rows. The median number of rows per baseline table was 15 and the median number of columns was 2. The central 50% of publication dates were between 15 June 2016 and 15 June 2020. The summary statistics extracted from the baseline tables of the included trials are in Table 4.

**Table 4.** Summary statistics from the baseline tables of *PubMed Central* trials. The first column indicates if the statistics were included in the tests of under- or over-dispersion.

Included	Statistic	n	Percent
Yes	Percent	37,229	52
	Continuous	27,539	38
	Number	2816	4
	Confidence interval	38	<1
No	Median	4212	6
	P-value	206	<1
	Range	50	<1
	Total	72,090	100

After excluding summary statistics that were medians and ranges, which cannot be compared using t-statistics, and excluding perfectly correlated table rows, there were 2245 trials with 52,615 table rows available for the Bayesian model. There were a relatively large number of trials that had baseline tables that were over-dispersed: 18.3% for the 0.95 threshold. There were fewer trials that were flagged as under-dispersed: 3.6% for the 0.95 threshold.

**Figure 2.**
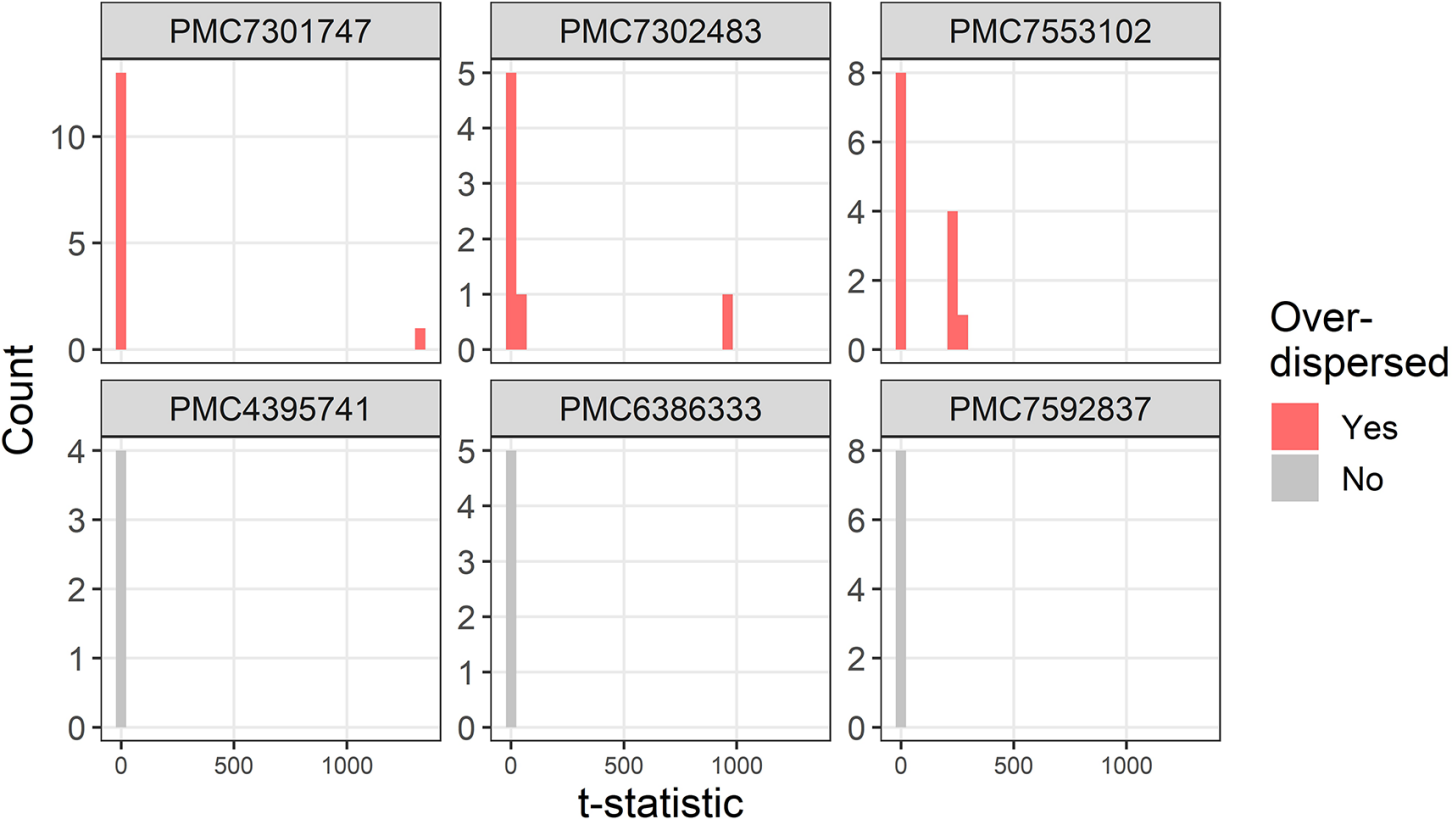
Example t-distributions for three trials flagged as over-dispersed. For comparison there are three randomly selected trials that were not flagged. The scales on the count axes differ by trial. The panel headings show the PubMed Central ID number.

The t-distributions for three trials that were flagged as over-dispersed ( ${\bar{P}}_{i}=1,{\bar{\epsilon }}_{i}<1$ ) are plotted in Figure 2. For comparison, three randomly selected trials with no dispersion ( ${\bar{P}}_{i}=0$ ) are also plotted. The three flagged trials were selected using the smallest multiplier of the precision ( ${\bar{\epsilon }}_{i}$ ) and hence show the most extreme over-dispersed trials. For each trial there are a small number of extremely large t-statistics.

Two trials that are flagged as over-dispersed are due to errors in the data extraction algorithm (PMC7553102 and PMC7301747). For PMC7553102 the bottom five rows of the table were wrongly assigned by the algorithm as continuous instead of percentages which creates t-statistics over 200. The result for PMC7301747 is an example of where an error in my data extraction creates a false impression of variability. The error occurs due to large numbers such as “15,170 (7,213)” which my algorithm extracts as three statistics: 15170, 7 and 213, instead of the correct two statistics: 15179 and 7213. This is because a comma is used both for large numbers and as a separator of two statistics such as a range. The t-statistic for this row is over 1,000 and hence the trial is flagged as over-dispersed.

The baseline table in trial PMC7302483 had a complex layout with four summary statistics per group in four separate columns, which the algorithm interpreted as separate groups rather than summary statistics for the same group.

**Figure 3.**
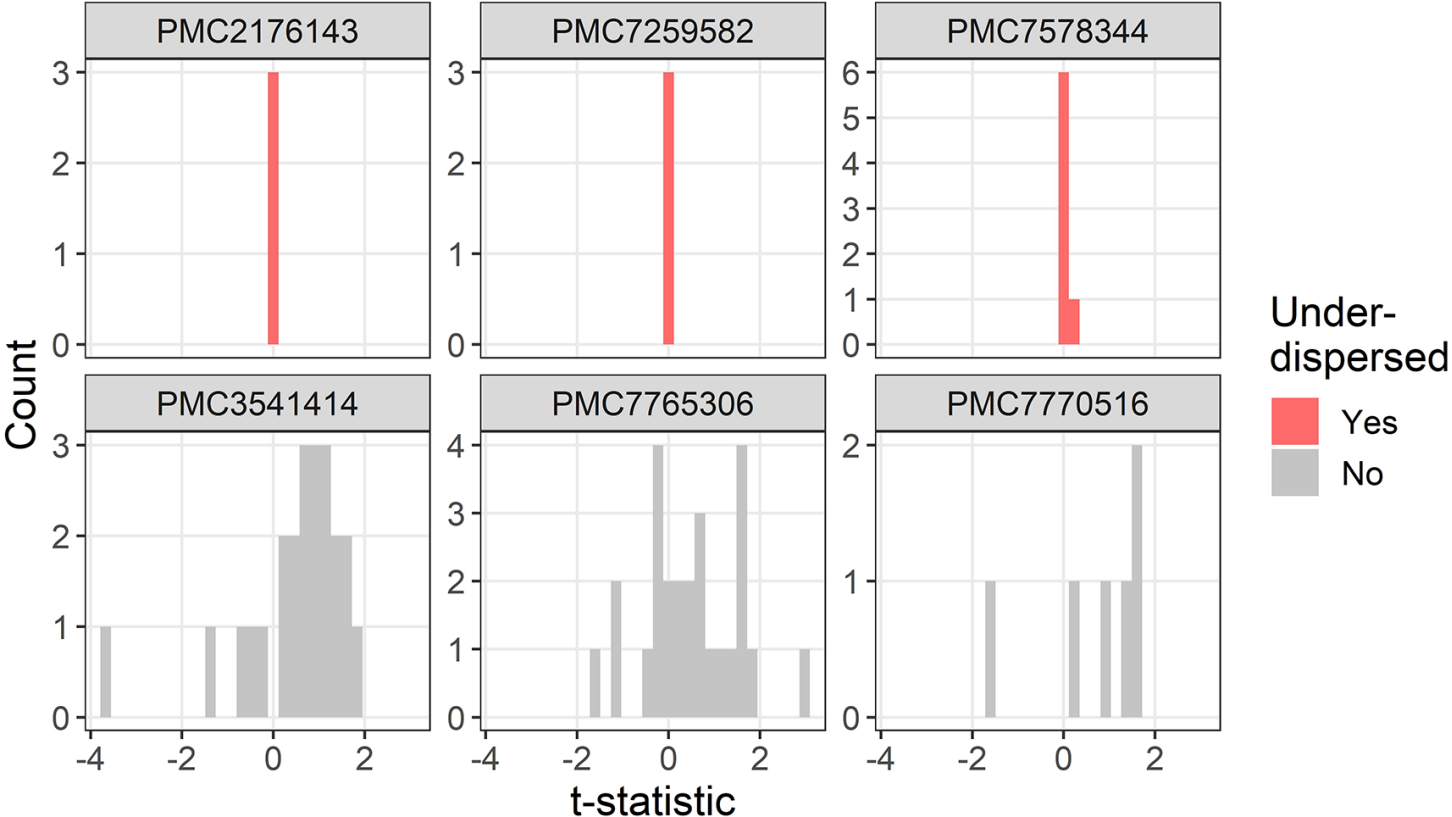
Example t-distributions for three trials flagged as under-dispersed. For comparison there are three randomly selected trials that were not flagged. The scales on the count axes differ by trial. The panel headings show the PubMed Central ID number.

The t-distributions for three trials that were flagged as under-dispersed ( ${\bar{P}}_{i}=1,{\bar{\epsilon }}_{i}>1$ ) are plotted in Figure 3. One study flagged as under-dispersed had an error where a mean was outside the confidence interval (PMC7259582), which meant the summary statistics were not recognised as a confidence interval and were instead wrongly guessed as a mean and standard deviation.

One flagged study was not a randomised trial but was a case–control study with an age and gender matched control group (PMC2176143), hence it was not surprising that the summary statistics in the baseline table were very similar.

One trial labelled proportions as percentages and hence it appeared as if there were lots of zero percentages which meant the two randomised groups appeared highly similar (PMC7578344).

The most extreme results in terms of under- and over-dispersion were often failures in the algorithm’s data extraction, sometimes due to poor reporting. Hence, I next examine less extreme results by excluding flagged trials that are in the tails of the precision distribution ( $\bar{\epsilon }$ ), which were the extremely under- or over-dispersed results (see extended data for the distribution). All flagged trials have a probability of under- or over-disperion of 1 ( ${\bar{P}}_{i}=1$ ).

**Figure 4.**
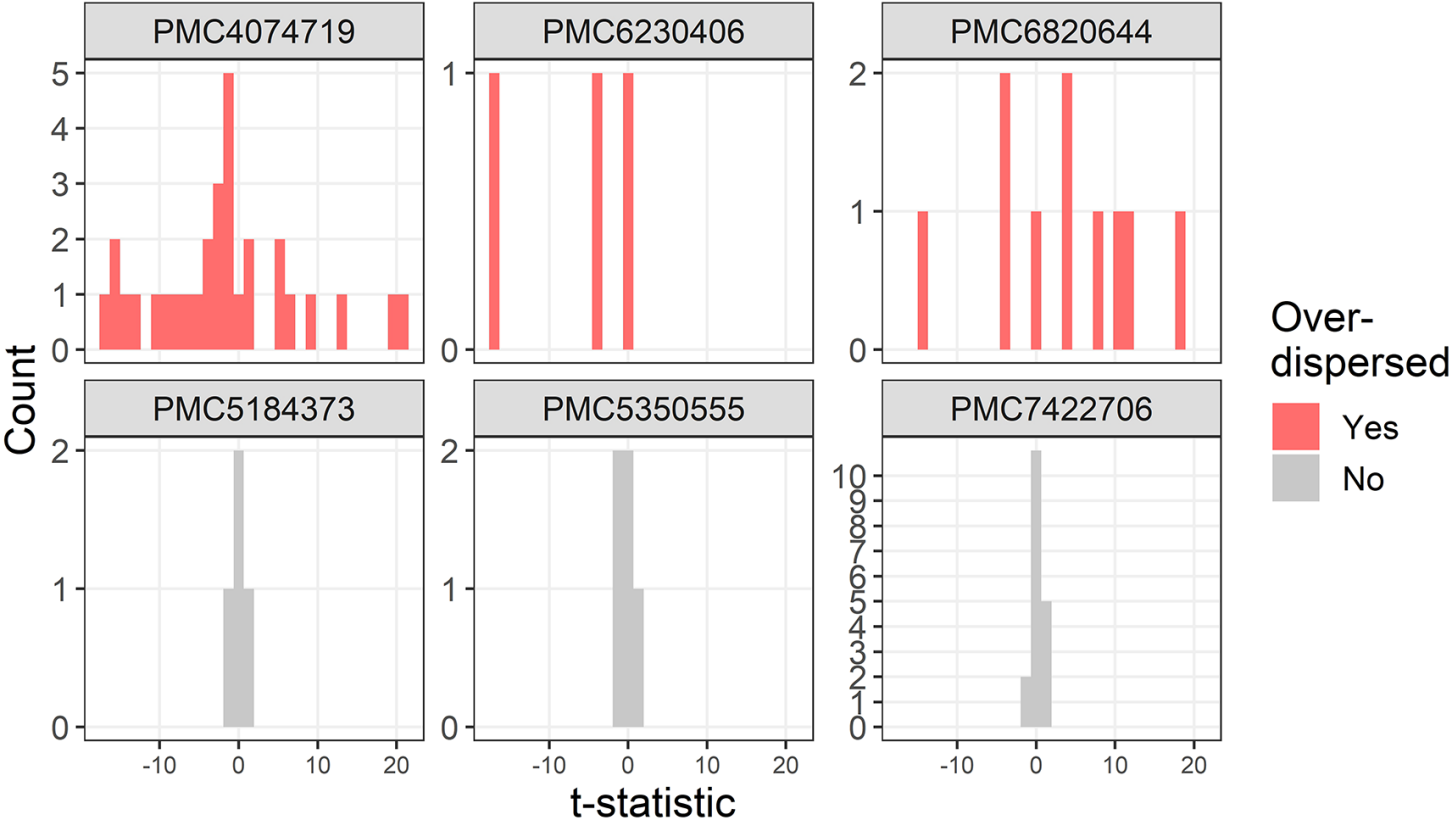
Example t-distributions for three trials flagged as over-dispersed after excluding the most extreme results. For comparison there are three randomly selected trials that were not flagged. The scales on the count axes differ by trial. The panel headings show the PubMed Central ID number.

Three further examples of over-dispersion are in Figure 4. One trial stratified the randomised groups by severity which created large between group differences and hence the over-dispersion (PMC4074719).

A trial that was flagged as over-dispersed had standard deviations for height that were zero (PMC6230406). This is likely a reporting error as zero standard deviations would require all participants to have exactly the same height.

One study was not a trial but was an observational study with some very large differences between groups at baseline, with 4 absolute t-statistics larger than 10, including a table row that was labelled as not significantly different based on a Mann–Whitney test but had a t-statistic of 19 (PMC6820644).

**Figure 5.**
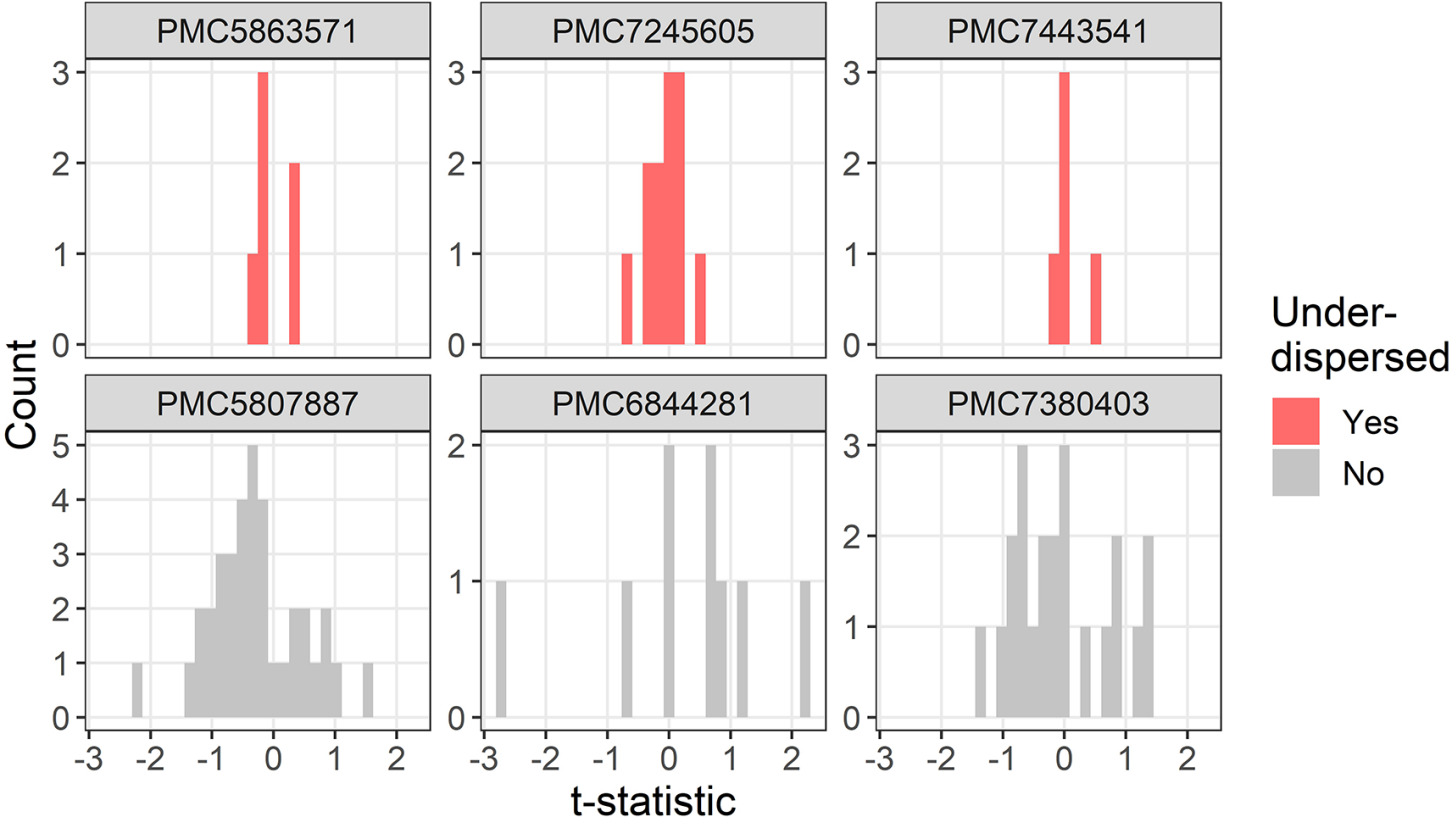
Example t-distributions for three trials flagged as under-dispersed after excluding the most extreme results. For comparison there are three randomly selected trials that were not flagged. The scales on the count axes differ by trial. The panel headings show the PubMed Central ID number.

Three examples for under-dispersion using the lower threshold of a multiplier are in Figure 5. All three trials have strikingly similar summary statistics, with all six t-statistics within –0.4 to 0.4 (PMC5863571), all twelve t-statistics within –0.7 to 0.5 (PMC7245605), and all five t-statistics within –0.2 to 0.6 (PMC7443541). One trial (PMC7443541) appeared to exclude two participants, potentially based on their baseline values, which may partly explain the under-dispersion.

### Predictors of under- or over-dispersion

I examined which study design features were associated with the trial-specific probability of under- or over-dispersion ( ${\bar{P}}_{i}$ ). The five predictors selected by the elastic net approach are in Table 5. The variables not selected were: number of table rows, pilot trial, block randomisation, average number of decimal places, journal, and first author’s country.

**Table 5.** Estimated predictors of the probability of under- or over-dispersion showing the mean change in probability and 95% confidence interval (CI).

Predictor	Mean	CI
Standard error	0.30	0.19 to 0.40
Difference in group sample sizes of 10+	0.13	0.09 to 0.16
Proportion continuous (0.0 to 1.0)	0.10	0.06 to 0.14
Number of table columns (+1)	0.09	0.07 to 0.11
Sample size (per doubling)	0.03	0.02 to 0.04

The probability of under- or over-dispersion was much higher in baseline tables that wrongly used the standard error of the mean instead of the standard deviation. This is as expected given that the standard error will be far smaller than the standard deviation and hence small differences could look like over-dispersion.

The probability of under- or over-dispersion increased when there were large differences in group sample sizes. An examination of examples of these trials found that some were not a simple comparison of, for example, treatment versus control (two columns), but included subgroups, such as gender or disease severity. These strata will likely create over-dispersion as the comparisons are no longer between randomised groups.

The probability of under- or over-dispersion increased when the baseline table had a greater proportion of continuous variables. This is likely because of the greater statistical power for continuous variables compared with categorical. Similarly the probability increased with greater sample size and more columns, which both increase the statistical power. The number of rows in the table was not selected, but in a separate simulation I confirmed that—as expected—the power to detect under-dispersion increased for larger tables (see extended data).

No journals or countries were selected by the elastic net variable selection, meaning none were associated with dispersion. However, the total number of trials were small for most journals and some countries, which reduces the statistical power. The largest number of trials for a single journal was 85.

## Discussion

### Statistical methods

In the simulation study, my Bayesian model based on the distribution of t-statistics outperformed the test using the distribution of p-values. My model dealt well with data that was skewed and categorical, or where the summary statistics were rounded, whereas the uniform test often wrongly flagged these trials as under- or over-dispersed.

The uniform test had a high false positive percentage because it is overly sensitive to small departures from the uniform distribution. Skewed or categorical data can cause spikes in the p-value distribution, causing the uniform test to be rejected even for randomised data. The Bayesian model using t-statistics is less sensitive to small departures as it examines the variance of the distribution, which is a summary statistic of the distribution rather than the entire distribution.

A previous simulation study similarly found that p-values in a baseline table for categorical data can be non-uniform even for trials that were randomised, and hence recommended against including categorical data. ^17^ My model using t-statistics can use continuous and categorical summary statistics, and as 56% of summary statistics in the *PubMed Central* data were numbers or percentages ( Table 4) this greatly increases the available data. The advantage of using both categorical and continuous summary statistics was shown in the example using known problematic trials, where the continuous-only results had lower probabilities and much greater uncertainty ( Figure 1).

### Automated extraction and testing of baseline tables

My automated algorithm was able to flag baseline tables that would be worth querying with the authors during journal peer review. However, this was not always due to under- or over-dispersion, but was sometimes because of an error in the table, because the authors had mislabelled their study as a trial, or because of the exclusion of valid data (PMC7443541). Flagging these issues with authors at the submission stage could reduce errors and improve reporting. Arithmetic and calculation errors were considered an important and common mistake by medical journal editors. ^30^

At times my algorithm flagged papers where the baseline table was not a baseline table for a randomised trial, but was a study that re-used the data from a trial. For example, a study which examined responders and non-responders to a randomised treatment, and the table compared non-randomised groups meaning over-dispersion is likely (PMC7660513). It is challenging to exclude these studies using automation as the abstract and title naturally talk about the randomised trial. Any automated flags raised for papers like this would need to be filtered by a person, or the authors whose study was flagged could simply explain that it was not a randomised trial. An automated algorithm to detect fake papers containing “tortured phrases” used a two-stage (or semi-automated) approach, where results that are flagged by an algorithm need to be checked by a person. ^31^

Publishers have trialled automated algorithms to check statistics and reporting completeness. ^32^^–^^34^ If applied by a publisher, my algorithm could be adapted to suit the publisher’s style; the current algorithm tried to cover all journals. The statistics that control which papers are flagged ( ${\bar{P}}_{i}$ and ${\bar{\epsilon }}_{i}$ ) could be tuned with experience to reduce false positives.

My algorithm flagged some trials that were under-dispersed with a striking similarity in the baseline characteristics of randomised groups. Flagging trials where the baseline table is under-dispersed might protect journals from publishing fraudulent papers, as this has been a clue in previous fraud investigations. ^35^ It is better to prevent the publication of fraudulent papers, as post-publication retractions can be long and costly. ^5^ A study of randomised trials submitted to the journal *Anaesthesia*, estimated that around one quarter of trials had false data that was problematic enough to invalidate the trial. ^5^ Research fraud may be increasing due to fierce competition for funding and promotion that often depend on publication counts. ^36^

### Improved reporting

There were many potential trials that were excluded because they were not randomised trials ( Table 3). Two key reasons for this were poor reporting in the title and abstract, ^37^ and studies that re-used data from a trial in other study designs (e.g., PMC6761647). Some baseline tables were excluded or flagged as under- or over-dispersed because of atypical descriptions in the caption or because of complex formatting in the table. There is a great variance across journals in how baseline tables are reported, including varied uses of symbols, labels and punctuation. Ideally commas would not be used to separate two numbers as they are also used to indicate thousands and millions.

I found many mistakes in baseline tables, some of which meant the trial was flagged as under- or over-dispersed. Mistakes included misreported statistics (e.g., continuous summary statistics labelled as percentages), missing labels, typographical errors, means reported without standard deviations, zero standard deviations, incorrect confidence intervals, incorrect p-values, and percentages that did not match the numerator divided by the denominator. Researchers should take more care and accurately report their results. ^38^

Greater use of standardised reporting—such as recommended by CONSORT—would increase the amount and accuracy of data that can be captured using automation. Even supposedly simple statistics such as age and gender were inconsistently presented in baseline tables, and a previous study similarly found highly varied reporting in age and gender in the *clinicaltrials.gov* database. ^29^ Publishers who wanted to use my algorithm to screen trials may need to provide more guidance to authors, although journal editors have raised concerns that authors rarely read instructions, ^39^ and there is no systematic study of whether journal instructions are read. ^40^

### Relation to previous work

Carlisle plotted the distribution of standardised mean differences for continuous summary statistics from randomised trials and graphically compared the distribution to a standard normal to visually check for under- or over-dispersion. ^41^

A number of automated algorithms have been created to detect numerical problems in papers, including *statcheck* for p-values ^42^ and *SPRITE* for summary statistics. ^43^ An automated check of p-values and confidence intervals found up to 5% had large errors, suggesting there are likely tens of thousands of published papers containing undetected errors. ^44^ These automated checks had a similar motivation: to automate the laborious process of checking numerical results and improve the quality of published papers and/or correct errors in published papers.

### Limitations and extensions

A previous study found that 92% of trials included a baseline table, ^1^ whereas my algorithm only extracted a baseline table for 25% of trials, hence I very likely excluded eligible trials where the algorithm did not detect a baseline table. Often this was because the baseline table was in graphical format meaning the table could not be extracted. There were other exclusions where the study was not a randomised trial, and hence no baseline table was included. There were also trials that did not include a baseline table (e.g., PMC3574512).

I assumed one sample size per group, but there were tables where the sample size varied by row (e.g., PMC7086156) and hence my calculated t-statistics will be inaccurate. Some trials had multiple baseline tables (e.g., PMC7908111), however I just used the first table. Some baseline tables were in an appendix and I only extracted tables from the main text in XML format.

In the simulation study, I assumed a that 50% of statistics were copied when the data were under-dispersed, but fraudsters may copy fewer statistics on average or use an entirely different process for falsifying data.

I report whether a baseline table has a potential problem, but make no attempt to differentiate between fraud and honest errors. ^35^^,^^45^ Checking for fraud needs to be done by examining other details, such as ethics clearances, plausible recruitment rates, and other work by the same authors. However, my automated check could still be a useful flag when papers are submitted. ^46^

I used t-statistics to test for issues in baseline tables, but other methods could be applied such as Benford’s law. ^35^^,^^47^

Statistical approaches have been used to detect fraud in trials using individual patient data. ^3^^,^^6^^,^^48^ Problems can be more accurately detected in individual data than summary statistics and this also avoids any rounding errors. ^5^^,^^6^ My approach could be extended to examine the dispersion in individual data at baseline, which would greatly increase its ability to detect under- or over-dispersion. ^5^ Journals could request that authors provide the underlying trial data at submission to perform detailed checks. ^49^^,^^50^ Authors may raise concerns about participant confidentiality and data security, ^51^ but many data sets in health collect anonymised data and authors need not commit to openly sharing their data or sharing any variables that include personal information.

I have created a *shiny* app where researchers can upload the summary statistics for a trial to examine the distribution of t-statistics and get the results from my Bayesian model https://aushsi.shinyapps.io/baseline/. This app should be useful for researchers who are concerned about particular papers.

## Conclusions

My automated algorithm is potentially useful as an initial screening of randomised trials, but needs human validation of the trials that are flagged as under- or over-dispersed as the automated data extraction is imperfect. Similar automated tools are likely to become more widely used as journals struggle to find reviewers due to increasing submission numbers and over-burdened reviewers. ^52^

## Data availability statement

### Underlying data

Zenodo: baseline tables. https://doi.org/10.5281/zenodo.6647853. ^53^

This project contains the following underlying data:

• simulated_data_bland.RData. The simulated trial data.
• trialstreamer.RData. The trials identified by *trialstreamer* and downloaded from *PubMed Central.*
• hand_entered_data.RData. The manually entered data used in the algorithm’s validation.

Data are available under the terms of the Creative Commons Attribution 4.0 International license.

### Extended data

• Github: “Supplement: Automated detection of over- and under-dispersion in baseline tables in randomised controlled trials” (baseline_tables_supplement.docx)
• Github: “Algorithm validation: Comparison of the statistics extracted from baseline tables by the algorithm and manually” (3_compare_algorithm_hand.docx)

This project contains the following extended data: https://doi.org/10.5281/zenodo.6647853.

## Reporting guidelines

Repository: STROBE checklist for “Automated detection of over- and under-dispersion in baseline tables in randomised controlled trials”. https://github.com/agbarnett/baseline_tables/tree/main/checklist.

## Author contributions

Adrian Barnett: Conceptualization, Investigation, Methodology, Software, Visualization, Writing - Original Draft.
