# Supplementary material for: Automated detection of over- and under-dispersion in baseline tables in randomised controlled trials
Source: F1000Res. 2023 May 30;11:783. Originally published 2022 Jul 12. [Version 2] doi: 10.12688/f1000research.123002.2 (PMC10285343; doi:10.12688/f1000research.123002.2)
Supplement: Peer review history

# Peer review: Reviewer response for version 2

**DOI:** 10.5256/f1000research.148868.r175858

**Published:** 2023-06-06

**Authors:** Erik van Zwet

**Affiliations:** 1 Department of Biomedical Data Sciences, Leiden University Medical Center, Leiden, The Netherlands

I’m satisfied with the author’s response to my comments.

Is the work clearly and accurately presented and does it cite the current literature?

Yes

If applicable, is the statistical analysis and its interpretation appropriate?

Partly

Are all the source data underlying the results available to ensure full reproducibility?

Yes

Is the study design appropriate and is the work technically sound?

Yes

Are the conclusions drawn adequately supported by the results?

Partly

Are sufficient details of methods and analysis provided to allow replication by others?

Yes

Reviewer Expertise:

I am a statistician at a university hospital.

I confirm that I have read this submission and believe that I have an appropriate level of expertise to confirm that it is of an acceptable scientific standard.

# Peer review: Reviewer response for version 1

**DOI:** 10.5256/f1000research.135061.r170574

**Published:** 2023-05-12

**Authors:** Jack Wilkinson

**Affiliations:** 1 Centre for Biostatistics, Faculty of Biology, Medicine, and Health, University of Manchester, Manchester, UK

The paper describes an automated approach to extracting baseline tables from RCTs and testing for under or over-dispersion. The work is timely, as there is currently lots of interest in and awareness around the issue of fabricated RCT data. Efficient methods to flag trials with apparent problems are needed – as the author notes, manual checking of published papers (as well as of the underlying individual participant data, if an investigation proceeds to that stage) is very time-consuming. My view of the work is positive – below I make some comments on applicability of the comparison between the new approach versus the approach based on uniformity of p-values.

- The uniform test has been applied to binary variables in the simulation study – as the author notes, this is incorrect – and we probably don’t need another simulation study to tell us this. A more useful comparison might have been to compare the t-statistic method (on all variables) to the uniform p-value method applied to continuous variables only. Do we miss any studies using the uniform method because we have to omit binary variables? As it stands, we appear to have a comparison between the new approach and the uniform p-value approach applied to variables which are not suitable. Since one of the possible strengths of the new method is that it allows binary variables to be inspected, it would have been useful to know whether including them (vs dropping them with the uniform p-value method) actually led to different results.
- Aside from the test of dispersion, the ability to reliably extract baseline table results would itself be very useful. This could be used to apply other checks (e.g. GRIM, checking accuracy of reported p-values, checks of digit preference) and if it could be done on a large scale, could facilitate identifying duplication between tables. So the ability of the tool to reliably extract this information is of interest. This appears to be challenging – tables were extracted from 25% of papers, when they will probably be reported by nearly all of them. Still, as someone who investigates a lot of papers for integrity issues, the ability to automatically extract for 1 in 4 papers would be welcome.
- The paper mentions ‘dynamic randomisation’ as a challenge to investigating dispersion – not sure what specifically was meant by this term, but any method for creating balance in baseline characteristics will cause issues here (stratified randomisation, minimisation). However, when looking at study features associated with dispersion, this sort of covariate-adaptive randomisation doesn’t appear to have been considered – blocked randomisation has been considered, but this balances group sizes, rather than covariates.
- As presented, results in Table 5 may be misleading (due to ‘Table 2 fallacy’ - Table 2 Fallacy: Presenting and Interpreting Confounder and Modifier Coefficients | American Journal of Epidemiology | Oxford Academic (oup.com)).
- Various limitations of the approach are helpfully documented by the author.
- The suggestion to focus on t-statistics is interesting, and is a useful contribution to the ongoing methodological work on this topic. There remains considerable scope to evaluate the approach, and to consider how it might sit alongside other approaches for integrity checking.`

Is the work clearly and accurately presented and does it cite the current literature?

Yes

If applicable, is the statistical analysis and its interpretation appropriate?

Yes

Are all the source data underlying the results available to ensure full reproducibility?

Yes

Is the study design appropriate and is the work technically sound?

Yes

Are the conclusions drawn adequately supported by the results?

Partly

Are sufficient details of methods and analysis provided to allow replication by others?

Yes

Reviewer Expertise:

Stats, trials, detection of inauthentic trials

I confirm that I have read this submission and believe that I have an appropriate level of expertise to confirm that it is of an acceptable scientific standard.

# Peer review: Reviewer response for version 1

**DOI:** 10.5256/f1000research.135061.r150544

**Published:** 2022-09-16

**Authors:** Erik van Zwet

**Affiliations:** 1 Department of Biomedical Data Sciences, Leiden University Medical Center, Leiden, The Netherlands

This paper addresses the important problem of the detection of fraudulent RCTs. The basic idea is to analyze the baseline table or “table 1” and test if the group differences are either too large or too small. The author cites previous work on this approach.

 The novelty of the paper is to propose a Bayesian model that can switch between the distribution of the differences between the groups that would be expected from chance alone, versus and over- or underdispersed distribution that would result from manipulation. For numerical outcomes the central t-distribution is appropriate when there is no fraud, and over- or underdispersion can be modelled by multiplying the dispersion with some trial-specific factor (gamma). Somewhat surprisingly, the t-distribution is also used for categorical outcomes with the author mentioning that the t-test is robust even for small sample sizes.

 A minor comment is that the prior probability of fraud is taken to be 50%, which seems rather pessimistic.

 I do have one serious reservation which led me to report that the statistical analysis and interpretation are "partly" appropriate. Although it is not mentioned explicitly, it seems that the Bayesian model assumes that the variables in the baseline table are independent, or at least uncorrelated. This does not seem realistic. Suppose the baseline table has, among other variables, age and blood pressure. If, by chance, a large number of old people are selected in group A, then it is likely that group A will also have a large number of people with high blood pressure.

 Independence certainly does not hold among the pairwise group differences when there are more than 2 groups. Now, I’m worried that the dispersion parameter will pick up on this dependence, which could result in the false detection of fraud.

 From the description of the simulation experiment, it seems that the variables were independently generated. It would be important to also examine scenarios with varying degrees of dependence.

 The result from the simulation study (p.7) that underdispersion was detected in only 16% of the cases is a little disappointing. Especially since underdispersion seems more likely than overdispersion as fraudsters presumably want their Table 1 to look beautifully balanced.

 Finally, I always recommend the paper of Morris et al., when I review a simulation study that does not cite it already ^1^.

Is the work clearly and accurately presented and does it cite the current literature?

Yes

If applicable, is the statistical analysis and its interpretation appropriate?

Partly

Are all the source data underlying the results available to ensure full reproducibility?

Yes

Is the study design appropriate and is the work technically sound?

Yes

Are the conclusions drawn adequately supported by the results?

Partly

Are sufficient details of methods and analysis provided to allow replication by others?

Yes

Reviewer Expertise:

I am a statistician at a university hospital.

I confirm that I have read this submission and believe that I have an appropriate level of expertise to confirm that it is of an acceptable scientific standard, however I have significant reservations, as outlined above.

## References

[1] : Using simulation studies to evaluate statistical methods. *Statistics in Medicine* .2019;38(11) : 10.1002/sim.8086 2074-2102 10.1002/sim.8086 PMC649216430652356

# Response

**Published:** 2022-09-23

**Authors:** Adrian Barnett

**Affiliations:** Queensland University of Technology, Australia

*A minor comment is that the prior probability of fraud is taken to be 50%, which seems rather pessimistic.*

 Response: Yes, this could be viewed as pessimistic. When considering a single trial, it has the least amount of prior information, because it gives equal probability to both scenarios: dispersion and no dispersion. However, there may be prior knowledge about the expected probability of dispersion which could be lower, say 0.10. I have updated the shiny app to allow users to give their own prior probability.

*I do have one serious reservation which led me to report that the statistical analysis and interpretation are "partly" appropriate. Although it is not mentioned explicitly, it seems that the Bayesian model assumes that the variables in the baseline table are independent, or at least uncorrelated. This does not seem realistic. Suppose the baseline table has, among other variables, age and blood pressure. If, by chance, a large number of old people are selected in group A, then it is likely that group A will also have a large number of people with high blood pressure.*

*Independence certainly does not hold among the pairwise group differences when there are more than 2 groups. Now, I’m worried that the dispersion parameter will pick up on this dependence, which could result in the false detection of fraud.*

*From the description of the simulation experiment, it seems that the variables were independently generated. It would be important to also examine scenarios with varying degrees of dependence.*

 Response: I have added three new simulations that examine correlated data. I simulated correlated continuous data, such as age and blood pressure, with separate simulations for correlation strengths of low (0.2) and high (0.6). I also added a simulation with 3 treatment arms. None of these simulations had over- or under-dispersion, so they were used to check for false positives. The Bayesian model did have an inflated false positive percentage for the high correlated data of 11%. However, it performed well for the 3-arm simulation, whereas the uniform test again had a high false positive probability of 23%.

 As per the recommended paper of Morris, it is: “useful to have unrealistically extreme data-generating mechanisms to understand when and how each method fails”. Trials with only multiple continuous variables with a correlation of 0.6 are not “unrealistically extreme”, but are likely rare. However, knowing that the approach can return false-positives in this scenario is useful to know, and I have added a comment on this to the discussion.

 Overall, I believe the new simulations show that the Bayesian method is relatively robust to correlated data.

*The result from the simulation study (p.7) that underdispersion was detected in only 16% of the cases is a little disappointing. Especially since underdispersion seems more likely than overdispersion as fraudsters presumably want their Table 1 to look beautifully balanced.*

 Response: I agree this is a disappointing result. This lack of power can be somewhat overcome by combining tables from the same author or research group, as per the example in Figure 1. I have also mentioned this disappointing result in the discussion.

 Over-dispersion is generally easier to detect, because it is unbounded, and even one row of the table with a large difference could mean a high probability of over-dispersion. Whereas under-dispersion is bounded at zero, and hence generally needs multiple table rows with small differences to increase the probability of under-dispersion.

*Finally, I always recommend the paper of Morris et al., when I review a simulation study that does not cite it already.*

 Response: Thanks for recommending this paper. I have heeded the advice of Morris et al. and included the standard error of the uncertainty in a supplemental plot and added some information justifying the sample size.
